# Diagnostik der Amyloidose im Rahmen der Früherkennung bei hand- und plastisch-chirurgischen Patienten – welches Gewebe sollte wie untersucht werden?

**DOI:** 10.1055/a-2739-3771

**Published:** 2025-11-27

**Authors:** Christoph Röcken

**Affiliations:** 1 Institut für Pathologie, Christian-Albrechts-Universität zu Kiel, Medizinische Fakultät, Kiel, Germany

**Keywords:** Amyloid, Kongorot, ATTR, Transthyretin, Karpaltunnelsyndrom, Pathologie, amyloid, Congo red, transthyretin, carpal tunnel syndrome, amyloid and amyloidoses

## Abstract

Amyloidosen sind eine heterogene Krankheitsgruppe, die sich durch die fibrilläre Ablagerung von Peptiden und Proteinen in einer β-Faltblattstruktur manifestieren. In Deutschland zählen die AL- und ATTR-Amyloidose zu den am häufigsten auftretenden systemischen Formen. Die ATTR-Amyloidose kennzeichnet der Befall des ligamentären und tenosynovialen Gewebes bevor es zu einer Herzbeteiligung kommt, die wiederum lebensbegrenzed sein kann. Dies führt zu einer signifikant erhöhten klinischen Relevanz der Diagnostik von ATTR-Amyloid in Resektaten von hand- und plastisch-chirurgischen Eingriffen. Zu den Erkrankungen, die in diesem Kontext als relevant erachtet werden, zählen das Karpaltunnelsyndrom, der schnellende Finger, die spontane Bizepssehnenruptur sowie die Spinalkanalstenose. Die Prävalenz des ATTR-Amyloids nimmt mit dem Alter zu und erreicht in der neunten Lebensdekade nahezu 50%. Die während des operativen Eingriffs gewonnenen Gewebeproben sollten in Formalin fixiert werden. Der Nachweis von Amyloid wird in der Regel durch die Kongorot-Färbung und unter Einsatz der Polarisationsmikroskopie erbracht. Die Sensitivität des Nachweises hängt von der Menge des gewonnenen Gewebes sowie dem jeweiligen Krankheitsstadium ab. Dabei ist zu berücksichtigen, dass jede Gewebeart und -probe das Risiko eines Stichprobenfehlers aufweist. Nach dem Nachweis von Amyloid ist eine Klassifizierung des Amyloids erforderlich, da Amyloidosen behandelbar geworden sind und die Therapie in Abhängigkeit vom Amyloidose-Typ erfolgt.

## Abstract (en)

Amyloidoses are a heterogeneous group of diseases characterised by the fibrillar deposition of peptides and proteins in a β-sheet configuration. In Germany, AL and ATTR amyloidosis are among the most common systemic forms. ATTR amyloidosis is characterised by the involvement of ligamentous and tenosynovial tissue prior to cardiac involvement, which, in turn, can be be life-limiting. This highlights the growing clinical relevance of diagnosing ATTR amyloid in resection specimens from hand and plastic surgery procedures. Diseases considered relevant in this context include carpal tunnel syndrome, trigger finger, spontaneous biceps tendon rupture, and spinal canal stenosis. The prevalence of ATTR amyloid increases with age, reaching nearly 50% in the ninth decade of life. Tissue samples obtained during surgery should be fixed in formalin. Amyloid can be detected using Congo red staining and polarisation microscopy. The sensitivity of detection depends on both the amount of tissue obtained and the stage of the disease. It should be noted that all tissue and sample types carry a risk of sampling error. Once amyloid has been detected, it must be classified, as amyloidoses are now treatable and treatment depends on the specific type of amyloidosis.

## Amyloid und Amyloidosen

Amyloid bezeichnet Peptide oder Proteine, die β-Faltblattfibrillen bilden und sich zu pathologischen, unlöslichen und toxischen Aggregaten zusammenlagern 1 . Ultrastrukturell sind diese Amyloidfibrillen unverzweigt und von variabler Länge mit einem Durchmesser von 7,5–10 nm 1 . Bis heute wurden 42 Peptide und Proteine identifiziert, die Amyloid bilden und sich lokal oder systemisch in verschiedenen Geweben und Organen sowohl intrazellulär als auch extrazellulär ablagern können ( Tab. 1 ) 2 . Der durch Amyloidablagerungen verursachte Krankheitszustand wird als Amyloidose bezeichnet und kann je nach Art des Amyloids und seiner histoanatomischen Verteilung zu unterschiedlichen klinischen Erscheinungsbildern führen. Amyloid wird histologisch mit der Kongorot-Färbung und Polarisationsmikroskopie diagnostiziert, wobei eine charakteristische anomale Polarisationsfarbe zu erkennen ist 1 . Anschließend wird das amyloidogene Protein mittels Immunhistochemie und/oder Massenspektrometrie (MS) identifiziert, was für die Wahl der geeigneten Behandlungsoptionen von entscheidender Bedeutung ist 3 .

**Table:** **Tab. 1** Übersicht über die im Jahr 2024 bekannten Amyloidproteine 2 .

Protein	Vorläuferprotein	S/L	E/H	betroffene Organe/ausgelöste Krankheiten
AL	Immunglobulinleichtkette (λ oder κ)	S, L	E, H	Alle Organe außer ZNS
AH	Immunglobulinschwerkette	S, L	E	Alle Organe außer ZNS
AA	(Apo) Serum-Amyloid-A	S	E, H	Alle Organe außer ZNS
ATTR	Transthyretin, Wildtyp (ATTRwt)	S	E	Herz vor allem bei Männern, Tenosynovialgewebe
	Transthyretin, Mutationsvarianten (ATTRv)	S	H	PNS, ANS, Herz, Glaskörper, Leptomeningen, Niere
Aβ2M	β2-Mikroglobulin, Wildtyp	S	E	Muskuloskelettales System
	β2-Mikroglobulin, Mutationsvarianten	S	H	ANS, Herz, Zunge
AApoAI	Apolipoprotein A-I, Mutationsvarianten	S	H	Herz, Leber, Niere, PNS, Hoden, Larynx, Haut
AApoAII	Apolipoprotein A-II, Mutationsvarianten	S	H	Niere
AApoAIV	Apolipoprotein A-IV, Wildtyp	S	E	Nierenmark, Knochenmark, Herz
AApoAIV	Apolipoprotein A-IV, Mutationsvarianten	S	E	Herz, Niere
AApoCII	Apolipoprotein C-II, Mutationsvarianten	S	H	Niere
AApoCIII	Apolipoprotein C-III, Mutationsvarianten	S	H	Niere
AGel	Gelsolin, Mutationsvarianten	S	H	PNS, Kornea, Niere
ALys	Lysozym, Mutationsvarianten	S	H	Niere
ALECT2	Leukozyten-chemotaktischer Faktor 2	S	E	v. a. Niere
AFib	Fibrinogen α, Mutationsvarianten	S	H	v. a. Niere
ACys	Cystatin C, Mutationsvarianten	S	H	PNS, Haut
ABri	ABriPP, Mutationsvarianten	S	H	ZNS
ADan*	ADanPP, Mutationsvarianten	L	H	ZNS
Aβ	Aβ Vorläuferprotein, Wildtyp	L	E	ZNS
	Aβ Vorläuferprotein, Mutationsvarianten	L	H	ZNS
AαSyn	α-Synuclein (αS)	L	E	ZNS
	α-Synuclein (αS), Mutationsvarianten	L	H	ZNS
ATau	Mikrotubuli-assoziiertes Protein τ	L	E	ZNS
	Mikrotubuli-assoziiertes Protein τ, Mutationsvarianten	L	H	ZNS
APrP	Prionenprotein, Wildtyp	L	E	Creutzfeldt-Jakob-Krankheit, Letale familiäre Insomnie
	Prionenprotein, Mutationsvarianten	L	H	Creutzfeldt-Jakob-Krankheit, Gerstmann-Sträussler-Scheinker-Syndrom, Letale familiäre Insomnie
	Prionenprotein, Mutationsvariante	S	H	PNS
ATMEM106B	Transmembrane 106B	L	E	Frontotemporale Lobärdegeneration
ACal	(Pro)Calcitonin	L	E	Medulläres Schilddrüsenkarzinom
	(Pro)Calcitonin	S	E	Niere
AIAPP	Amylin (Inselamyloidpolypeptid)	L	E	Langerhans‘sche Inselzellen, Insulinom
AANP	Atriales natriuretisches Peptid	L	E	Herzvorhof
APro	Prolaktin	L	E	Prolaktinom, Hypophyse im Alter
ASom	(Pro)Somatostatin	L	E	Somatostatinom
AGluc	Glukagon	L	E	Glukagonom
APTH	Parathormon	L	E	Nebenschilddrüsenadenom, Nebenschilddrüse im Alter
AIns	Insulin	L	E	Iatrogen, lokale Injektionen
AEnf	Enfuvirtide	L	E	Iatrogen, lokale Injektionen
AGLP1	Glukagon-ähnliches Peptid-1 -Analogon	L	E	Iatrogen, lokale Injektionen
AIL1RAP	Interleukin-1 Rezeptorantagonist	L	E	Iatrogen, lokale Injektionen
ASPC	Surfactant-assoziiertes Protein C (SP-C)	L	E	Lunge
ACor	Korneodesmosin	L	E	Verhorntes Plattenepithel, Haarfollikel
AMed	Laktadherin (MFG-E8)	L	E	Senile Aorta, Media der Gefäße
AKer	Keratoepithelin	L	E	Kornea, hereditär
ALac	Laktoferrin	L	E	Kornea
AOAAP	Odontogenes Ameloblasten-assoziiertes Protein	L	E	Odontogene Tumoren
ASem1	Semenogelin 1	L	E	Samenblasen
ACathK	Cathepsin K	L	E	Tumor-assoziiert
AEFEMP	*EGF-containing fibulin-like extracellular matrix protein 1*	L	E	Venen, altersassoziiert

S=systemisch, L=lokal, E=erworben, H=hereditär, ZNS=Zentrales Nervensystem, PNS=Peripheres Nervensystem, ANS=Autonomes Nervensystem. *ADan ist das gleiche Genprodukt wie bei ABri

## Physiologie

Die häufigste systemische Form der Amyloidose wird durch die Ablagerung des Amyloidproteins Transthyretin (ATTR) verursacht. Transthyretin (TTR) ist ein Plasmatransportprotein, das hauptsächlich von der Leber und in geringerem Maße vom Plexus choroideus und der Retina synthetisiert wird. Das Gen befindet sich auf Chromosom 18 (18q12.1), hat vier Exons und drei Introns und kodiert ein tetrameres Protein mit einer Molekülmasse von 55 kDa, das aus vier 127 Aminosäuren langen Monomeren besteht 4 . Das Monomer lagert sich zu einer β-Sandwich-Struktur zusammen, die aus einer kleinen α-Helix und acht β-Strängen besteht. Im Serum bildet TTR einen Proteinkomplex aus vier identischen Untereinheiten (Homotetramer) und ist für den Transport des Retinol-bindenden Proteins, des Vitamin-A-Komplexes sowie des Schilddrüsenhormons Thyroxin verantwortlich. Darüber hinaus wird TTR mittlerweile auch mit anderen zellbiologischen Funktionen in Verbindung gebracht, wie z. B. Neuroprotektion, Nervenregeneration, Zellschicksal, Proliferation und Immunregulation, metabolische Reprogrammierung und vielen anderen. Transthyretin gilt auch als zinkabhängige Metalloprotease 5 . Bei der ATTR-Amyloidose wird das TTR-Homotetramer destabilisiert und zerfällt in Monomere, die eine strukturelle Umwandlung durchlaufen und sich zu ATTR-Fibrillen neu zusammenlagern 4 .

Neben der ATTR-Amyloidose zählt die AL-Amyloidose zu den häufigsten systemischen Formen der Amyloidosen. Bei dieser Form der Amyloidose bilden die variablen Regionen der Leichtketten kappa und lambda das Amyloidprotein. Pathophysiologisch liegen der Erkrankung häufig eine monoklonale Gammopathie unklarer Signifikanz oder plasmozytisch differenzierte B-Zell-Neoplasien zugrunde 6 . Dabei lassen sich unterschiedliche chromosomale Aberrationen in den betroffenen Zellpopulationen nachweisen wie zum Beispiel Translokationen, Trisomien, chromosomale Zugewinne, Monosomien und Deletionen 7 . Die systemische Form der AL-Amyloidose hat im Allgemeinen eine schlechtere Prognose als die ATTR-Amyloidose. Daneben gibt es jedoch auch noch lokale Formen der AL-Amyloidose die im Gegensatz zur systemischen Form eine deutlich bessere Prognose haben 8 . Sie können jedoch durch raumforderndes Verhalten und Blutungen durchaus zu erheblichen klinischen Beschwerden führen.

## Klinik und Epidemiologie

Die ATTR-Amyloidose tritt in zwei verschiedenen Formen auf: der nicht-hereditären ATTR-Amyloidose (ATTRwt) ohne Keimbahnmutationen und mit Wildtyp-TTR als Vorläuferprotein und der hereditären ATTR-Amyloidose (ATTRv), die durch *TTR* -Keimbahnmutationen verursacht wird 2 .

Die Inzidenz von ATTRwt steigt mit zunehmendem Alter stetig an, wobei das Durchschnittsalter zum Zeitpunkt der Diagnose bei 77,1 Jahren liegt 9 . Die Inzidenzrate pro 100.000 Personen pro Jahr stieg von 1,50 (95% KI, 0,84–2,47) im Zeitraum 2006–2009 auf 4,92 (95% KI, 3,46–6,78) im Zeitraum 2016–2018, was teilweise auf ein gesteigertes Bewusstsein für die Krankheit zurückzuführen ist 10 . Männer sind häufiger betroffen als Frauen 9 . Die meisten ATTR-Herzamyloidosen (ATTR-CM) sind durch ATTRwt-Ablagerungen gekennzeichnet. Sie können klinisch symptomfrei sein oder aufgrund einer restriktiven Kardiomyopathie lebensbedrohlich sein. Kristen et al. haben gezeigt, dass die Menge an ATTR-Amyloid (sowohl ATTRwt als auch ATTRv) im Herzen mit dem Schweregrad der restriktiven Kardiomyopathie korreliert 11 .

Die häufigste Form der hereditären Amyloidose ist bei weitem ATTRv. Über 120 verschiedene Mutationen, die zu ATTRv führen, wurden identifiziert. Die Variante TTRV30M (p.TTRV50M) ist eine der häufigsten in Europa 12 . Die Prävalenz von ATTRv variiert je nach Symptomatik. Bei Patienten mit Polyneuropathie sind weltweit 10.000 Menschen betroffen, wobei ATTRv in Schweden, Japan und Portugal endemisch ist und dort eine Prävalenz von 1:1.000 aufweist. In diesen Ländern sind etwa 40.000 Patienten von ATTR-CM betroffen 12 . Klinisch dominiert entweder eine distale symmetrische sensomotorische Polyneuropathie [familiäre Amyloid-Polyneuropathie (FAP), insbesondere mit der Variante p.TTRV50M] oder eine Herzbeteiligung. Das autonome Nervensystem ist häufig betroffen, und es kann zu Malabsorption und schwerem Gewichtsverlust kommen. Das Erkrankungsalter liegt bei der *early onset* -Form zwischen 30 und 50 Jahren und bei der *late onset* -Form zwischen 60 und 80 Jahren. Unbehandelt verläuft die Krankheit oft innerhalb von zehn Jahren tödlich 13 .

Die ATTR-CM hat aufgrund der Entwicklung einer Herzinsuffizienz schwerwiegende Auswirkungen auf die Lebensqualität der Patienten und kann die Überlebenszeit auf etwa 4 Jahre verkürzen 12 . Verschiedene orthopädische Erkrankungen wie Karpaltunnelsyndrom, Spinalkanalstenose, atraumatische Bizepssehnenruptur und schnellender Finger können ATTR-CM um 5–10 Jahre vorausgehen und gelten als Warnsymptome 14 . Diese Erkenntnisse sind zum Thema von Fortbildungen für Kliniker geworden, da insbesondere die ATTRwt-Amyloidose heutzutage nicht mehr als seltene Erkrankung, sondern als endemische Krankheit gilt. Darüber hinaus ist die korrekte Zuordnung dieser Warnsymptome entscheidend für eine frühzeitige Diagnose und damit für eine erfolgreiche Therapie.

## Behandlung

Je nach betroffenem Organsystem ist die ATTR-Amyloidose mittlerweile behandelbar. Es wurden eine Reihe von Arzneimitteln entwickelt, die entweder das Homotetramer stabilisieren (Acoramidis, Diflusinal, Tafamidis) oder die Gentranskription oder -translation mithilfe von Antisense-Oligonukleotiden (Eplontersen) oder RNA-interferierenden Medikamenten (Patisiran, Vutrisiran) hemmen. In ausgewählten Fällen mit fortgeschrittener Amyloidose werden Herztransplantationen durchgeführt 15 16 17 . Antikörper, die die Clearance bereits gebildeter Amyloidfibrillen beschleunigen, befinden sich derzeit in klinischen Studien 18 .

Die systemische Form der AL-Amyloidose erfordert eine hämatoonkologische Betreuung und ist in der Regel zeitnah nach Diagnosestellung zu beginnen und durchzuführen, da jede Therapieverzögerung die Überlebenschancen der Patienten verschlechtert. Grundlegende Zielstruktur der Behandlung der AL-Amyloidose ist die plasmozytisch differenzierte B-Zelle/Plasmazelle, die das aberrante Protein sezerniert. Hierbei kommen unterschiedliche Therapien zum Einsatz wie zum Beispiel Bortezomib-Cyclophosphamid-Dexamethason mit und ohne Daratumumab, oder Hochdosis-Melphalantherapie mit nachfolgender autologer Stammzelltransplantation 19 20 .

### Gewebebasierte Diagnostik

Grundsätzlich kann Amyloid in jeder amyloidhaltigen Gewebeprobe nachgewiesen werden 3 . Dabei lassen sich drei verschiedene Szenarien unterscheiden.

Erstens, es besteht kein klinischer Verdacht auf das Vorliegen einer Amyloidose. Hier ist meist der Pathologe/die Pathologin der/die erste, die den Verdacht auf das Vorliegen von Amyloidablagerungen aufgrund eines auffälligen histologischen Befundes durch Anwendung der Kongorotfärbung und Polarisationsmikroskopie sichert.

Zweitens, es besteht ein klinischer Verdacht auf das Vorliegen einer Amyloidose und es wird gezielt eine Gewebeprobe entnommen zum Nachweis/Ausschluss einer Amyloidose. Für die Wahl der richtigen Gewebeprobe/Biopsienahmestelle muss die differenzialdiagnostisch in Erwägung gezogene Form der Amyloidose berücksichtigt werden 3 . Besteht der Verdacht auf das Vorliegen einer systemischen AL-Amyloidose, so eignet sich grundsätzlich eine Bauchfett- oder Bauchhautbiopsie zur Diagnosesicherung. Besteht der Verdacht auf das Vorliegen einer ATTRwt-Amyloidose, sind hier Bauchfettbiopsien zur Diagnosesicherung weniger geeignet, da sich ATTRwt-Amyloid seltener in Bauchfettbiopsien nachweisen lässt 21 22 . Hier sollte dann eher eine tiefe Rektumbiopsie erwogen werden in dem Sinne, dass sicher Submukosaanteile im Biopsat enthalten sind, um die dort häufig befallenen Gefäße zu erfassen 3 21 23 .

Drittens, es wird eine histologische „Routineuntersuchung“ durchgeführt an einer Gewebeart, in der häufig Amyloid als Zufallsbefund ohne unmittelbaren klinischen Verdacht nachweisbar ist. Hierzu zählen insbesondere die bei einer operativen Versorgung des Karpaltunnelsyndroms, der Spinalkanalstenose, des schnellenden Fingers oder einer spontanen Bizepssehnenruptur gewonnenen Gewebeproben.

## Feingeweblicher Nachweis von Amyloid

Die Kombination der Kongorotfärbung mit der Polarisationsmikroskopie wird nach wie vor als Goldstandard für den feingeweblichen Nachweis des Amyloids betrachtet. Es hat sich bislang kein anderes histochemisches und/oder lichtoptisches Verfahren durchgesetzt, dass diese Kombination flächendeckend in der mittelbaren, gewebebasierten Krankenversorgung ersetzt 3 . Der elektronenmikroskopisch geführte Nachweis der nicht verzweigten, starren Fibrillen variabler Länge ist für die Diagnose von Amyloid gleichwertig. Allerdings findet die Elektronenmikroskopie nur noch in seltenen Ausnahmefällen Anwendung, insbesondere in der Nierenbiopsiediagnostik, und ist somit kein breit eingesetztes Routineverfahren.

**Abb. 1:**
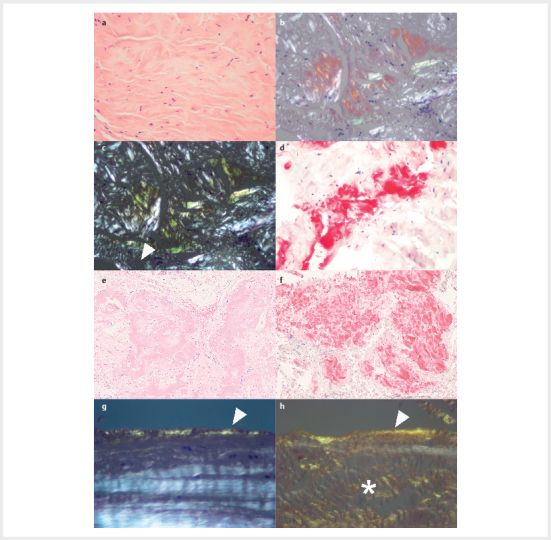
Amyloidablagerungen im Ligamtenum carpi transversum, der Bizepssehne und im Ringbandexzisat.Im Hämatoxylin-Eosin-gefärbten Schnittpräparat ( **a** ) eines Resektates vom Ligamentum carpi transversum können Amyloidablagerungen morphologisch unauffällig sein. In der Kongorot-Färbung zeigt sich die charakteristische anomale Polarisationsfarbe ( **b, c** ), die bei Rotation des Polarisationsfilters einen Farbumschlag von rötlich bis grün-gelblich zeigt (=anomale Polarisationsfarbe) und diagnostisch für das Vorliegen von Amyloid ist. Die anschließende immunhistologische Klassifikation weist ATTR-Amyloid nach ( **d** ). Ein Resektat einer spontanen Bizepssehnenruptur ( **e** ) enthält reichlich ATTR-Amyloid ( **f** ). Resektate vom Ringband beim schnellenden Finger enthalten oft entlang der Oberfläche des Rindenbandes bandförmige Amyloidablagerungen ( **g, h** : Pfeilspitze), bei denen es sich um eine lokale Form des AFib-Amyloids handelt 32 . Gleichzeitig können in der Matrix, unterhalb der Oberfläche, ATTR-Amyloidablagerungen auftreten (h – Asterisk), sodass dann eine echte Doppeltamlyoidose vorliegt. Hämatoxylin-Eosin ( **a, e** ); Kongorot in der Polarisationsmikroskopie ( **b, c, g, h** ); Anti-Transthyretin-Antikörper ( **d, f** ). Originalvergrößerungen 100-fach ( **e, f** ), 200-fach ( **a-d** ) und 400-fach ( **g, h** ).

Für die feingewebliche Diagnostik wird die Anwendung der Kongorotfärbung und Polarisationsmikroskopie der Nachweis einer charakteristischen anomalen Polarisationsfarbe gefordert 3 . Es handelt sich um ein Farbspiel, das von gelbgrünlich bis rotorange changiert ( Abb. 1 ). Die in der Literatur häufig verwendete Beschreibung der Polarisationsfarbe von „apfelgrün“ ist für die adäquate Beschreibung des komplexen Farbspektrums der anomalen Polarisationsfarbe unzureichend 24 . Die Polarisationsfarbe zeigt zudem unterschiedliche Intensitäten, was gelegentlich zu falsch negativen Befunden führen kann, wenn die Färbemethode und die Polarisationsmikroskopie nicht optimal aufeinander abgestimmt sind. In der Literatur wurden z. B. zwei verschiedene Varianten der anomalen Polarisationsfarbe bei der ATTR-Amyloidose beschrieben (1) eine schwache Affinität zum Kongorot, bei der überwiegend Fragmente des TTR in der Amyloidfibrille nachweisbar sind, und (2) eine stärkere Affinität zum Kongorot, bei der überwiegend intaktes, nicht proteolytisch gespaltenes TTR in der Amyloidfibrille abgelagert wird 25 .

Des Weiteren ist zu bedenken, dass der Nachweis von Amyloid mit dem Risiko eines Stichprobenfehlers verbunden ist. In einem frühen Krankheitsstadium kann es zu einem negativen Befund kommen, sofern das Amyloid nicht in der Gewebeprobe enthalten ist. Diese Aussage trifft auf Bioptate sämtlicher Gewebe und Organe zu, die einer Amyloiddiagnostik zugeführt werden, einschließlich tenosynovialen und ligamentären Gewebes. Es gibt keine Studien, die das Risiko des Stichprobenfehlers für tenosynoviale und ligamentäre Gewebeproben beziffern.

Eine weitere Einflussgröße ist die variable anatomische Verteilung des Amyloids: In einer eigenen Studie konnte gezeigt werden, dass beim Karpaltunnelsyndrom im Ligamentum carpi transversum signifikant mehr Amyloid enthalten ist als in der Synovialis 26 . Unsere Kohorte, bei der amyloidhaltiges Gewebe sowohl aus dem Ligamentum carpi transversum als auch von der Synovialis vorlagen, umfasste 32 Patienten 26 . Die Gesamtgröße der Studienkohorte betrug 4990 Gewebeproben von denen 582 (11,7%) ATTR-Amyloid enthielten 26 . Folgestudien kommen zu unterschiedlichen Ergebnissen bei kleineren Kohortengrößen. Ozdag et al. untersuchten eine Kohorte von 148 Patienten und fanden Amyloid in 21% (Synovialis) bzw. 22% (Ligamentum carpi transversum) der Fälle ohne den Amyloidtyp und den Amyloidgehalt zu bestimmen 27 . Navarro-Saez et al. untersuchten eine Kohorte von 246 Patienten und fanden Amyloid in 12,6% (Synovialis) bzw. 11,4% (Ligamentum carpi transversum) der Fälle ebenfalls ohne den Amyloidgehalt zu bestimmen 28 . Demgegenüber verglichen Elzinga et al. in einer retrospektiven Studie den Amyloidgehalt im Ligamentum carpi transversum mit demjenigen in der Synovialis und fanden keinen Unterschied. Die Kohorte umfasste allerdings nur 13 Patienten mit ATTRwt-Amyloid 29 .

Zahlreiche Studien belegen, dass der Nachweis von ATTR-Amyloid außerdem Alters- und Geschlechts-assoziiert ist. Amyloid findet sich häufiger bei Männern als bei Frauen (s. o.). Dabei steigt die Prävalenz kontinuierlich mit dem Alter an und erreicht ihren Gipfel im achten und neunten Lebensjahrzehnt 26 .

## Klassifikation des Amyloids

Wenn Amyloid im Gewebe nachgewiesen worden ist, muss es klassifiziert werden. Die Klassifikation dient der Zuordnung zur Grunderkrankung und ist entscheidend für die Prognose und Therapieplanung 3 . Dabei treten nicht alle 42 verschiedenen Formen von Amyloid in jedem Gewebe auf, sondern sie zeigen große Unterschiede in der Häufigkeitsverteilung und ihrer Zuordnung zu Gewebearten 2 . Einige Formen sind große Raritäten (insbesondere hereditäre Amyloidosen) oder nur auf bestimmte Organe begrenzt (zum Beispiel Zentralnervensystem, einige tumorassoziierte Amyloidosen oder das Amyloid der Samenblasen) ( Tab. 1 ). Von großer Relevanz bei tenosynovialen und ligamentären Geweben ist die Unterscheidung zwischen einer ATTR- und AL-Amyloidose. Die AA-Amyloidose ist hier eine Rarität und wird meistens eher in Nierenbiopsaten nachgewiesen, z. B. bei der Abklärung einer Proteinurie 30 .

Die ATTR-Amyloidose ist inzwischen der am häufigsten nachgewiesene Amyloidtyp in tenosynovialen und ligamentären Geweben und nach eigenen Auswertungen des Amyloidregisters in über 99% der Fälle nachweisbar (unveröffentlichte Beobachtung). Bei dem Ligamentum flavum findet sich außerdem noch häufiger Apolipoprotein A1 31 . Bei Ringbandexzisaten finden sich häufig zwei verschiedene Formen des Amyloids nicht selten auch zusammen im selben Präparat als sogenannte Doppeltamyloidose nämlich ATTR- und ein lokales AFib-Amyloid 32 . Deshalb muss bei Ringbandexzisaten stets eine korrekte Zuordnung der Immunreaktion zum Kongorot-gefärbten Serienschnitt erfolgen, um das Vorliegen einer Doppeltamyloidose nicht zu übersehen 32 .

Die Massenspektrometrie wird gern als sogenannter Goldstandard angeführt 3 . Allerdings birgt sie das Risiko, dass sie bei sehr geringer Ausdehnung des Amyloids nicht die notwendige Sensitivität aufweist und somit zu keinem Klassifikationsergebnis führen kann. Weiterhin fehlt ihr die räumliche Zuordnung zum Kongorot-gefärbten Schnittpräparat, die eine besondere Stärke der Immunhistologie ist. Außerdem ist die Massenspektrometrie zeitaufwendig und wird in Deutschland von den Kostenträgern nicht erstattet.

## Prädiktiver Wert des Amyloidnachweises

Epidemiologische Studien haben eindrückliche Belege geliefert, dass das Karpaltunnelsyndrom ein Prädiktor für Herzkrankheit und kardiale Amyloidose ist.

Eine Auswertung des nationalen dänischen Registers für den Zeitraum von 1996 bis 2012 ergab, dass 56.032 Patienten einer operativen Versorgung des Karpaltunnelsyndroms unterzogen worden waren 33 . Der Vergleich mit einer nach Alter und Geschlecht ausgewählten Vergleichskohorte zeigte dann, dass bei Patienten mit einem Karpaltunnelsyndrom im weiteren Verlauf signifikant häufiger eine Amyloidose diagnostiziert wurde als in der Vergleichsgruppe [Hazard Ratio: 12,12 (95% CI: 4,37 bis 33,60)]. Des Weiteren wurde bei Patienten, die an einem Karpaltunnelsyndrom litten, eine signifikant höhere Inzidenz einer Herzinsuffizienz beobachtet (Hazard Ratio: 1,54 (95% CI: 1,45 bis 1,64)). Darüber hinaus traten Vorhofflimmern, atrioventrikuläre Schenkelblockaden (AV-Block) und die Implantation eines Herzschrittmachers gehäuft auf 33 .

Eine landesweite Studie aus Schweden gelangt zu ähnlichen Resultaten. Bei Patienten, die an einem Karpaltunnelsyndrom litten, trat signifikant häufiger eine Herzinsuffizienz auf 34 . Gleiches gilt für die Spinalkanalstenose 34 35 . Eine kürzlich publizierte Metaanalyse kommt zu dem Ergebnis, dass bei 5–20% der Patienten mit Karpaltunnelsyndrom und histologisch gesichertem Nachweis von Amyloid im weiteren Verlauf eine kardiale Beteiligung auftritt 36 . Die feingewebliche Untersuchung von Resektaten im Rahmen des Karpaltunnelsyndroms ist von prädiktiver Bedeutung, da sie eine Frühdiagnostik der kardialen Amyloidose ermöglicht.

Da Amyloid nicht nur im Karpaltunnelresektat nachgewiesen wird, sondern auch in Resektaten bei Spinalkanalstenose (Ligamentum flavum) 37 , schnellendem Finger (Ringband) 32 und spontaner Bizepssehnenruptur 38 sollten auch die im Rahmen der operativen Versorgung diese Erkrankungen gewonnenen Gewebeproben einer feingeweblichen Untersuchung zugeführt werden. Auch wenn es bislang keine internationalen Leitlinien-Empfehlungen gibt, so zeigen zumindest die persönlichen Erfahrungen, dass ab dem 50. Lebensjahr der Patienten mit Amyloid zu rechnen ist 26 .

## Fazit für die Praxis

- Amyloid und Amyloidosen sind inzwischen behandelbare Erkrankungen.
- Der Nachweis von ATTR-Amyloid in Gewebeproben tenosynovialer und ligamentärer Herkunft nimmt mit dem Alter zu.
- Bei folgenden Erkrankungen findet sich regelhaft ATTR-Amyloid: Karpaltunnelsyndrom, schnellende Finger, spontane Bizepssehnenruptur, Spinalkanalstenose.
- Der Nachweis von Amyloid unterliegt grundsätzlich einem Stichprobenfehler und ist abhängig von der entnommenen Gewebemenge.
- Bei Karpaltunnelsyndrom findet sich Amyloid sowohl im Ligamentum carpi transversum als auch in der Synovialis.
- In Ringbandexzisaten treten oft zwei verschiedene Formen von Amyloid auf (AFib- und ATTR-Amyloid) im Sinne einer Doppeltamyloidose.
- Die Kongorotfärbung ist ein Goldstandard in der gewebebasierten Diagnostik und bei Nachweis von Amyloid muss anschließend eine Klassifikation des Amyloids erfolgen.
- Der Nachweis von ATTR-Amyloid weist einen gesicherten prädiktiven Wert für eine spätere Herzerkrankung auf. Dabei muss zum Zeitpunkt des Amyloidnachweises im Resektat noch keine kardiale Beteiligung vorliegen. Diese tritt unter Umständen mit einer Verzögerung 5-10 Jahren auf.
